# Hypertension and orthostatic hypotension in the elderly: a challenging balance

**DOI:** 10.1016/j.lanepe.2024.101154

**Published:** 2024-12-03

**Authors:** Julia Wiersinga, Sofie Jansen, Mike J.L. Peters, Hanneke F.M. Rhodius-Meester, Marijke C. Trappenburg, Jurgen A.H.R. Claassen, Majon Muller

**Affiliations:** a Department of Internal Medicine, Section Geriatrics, Amsterdam UMC, Boelelaan 1117, Amsterdam, the Netherlands; b Amsterdam Cardiovascular Sciences, Atherosclerosis & Ischemic Syndromes, Amsterdam, the Netherlands; c Department of Internal Medicine Section Geriatrics, UMC Utrecht, University of Utrecht, Utrecht, the Netherlands; d Alzheimer Center Amsterdam, Department of Neurology, Amsterdam UMC, Vrije Universiteit Amsterdam, Amsterdam Neuroscience, Amsterdam, the Netherlands; e Department of Geriatric Medicine, Oslo University Hospital, Ullevål, Oslo, Norway; f Department of Internal Medicine, Amstelland Hospital, Amstelveen, the Netherlands; g Radboud University Medical Center, Departments of Geriatrics, Radboud Research Institute for Medical Innovation and Donders Institute, Nijmegen, the Netherlands; h Department of Cardiovascular Sciences, University of Leicester, UK

**Keywords:** Hypertension, Orthostatic hypotension, Older patients

## Abstract

Hypertension and orthostatic hypotension (OH) frequently coexist in the older population, both stemming from impaired blood pressure (BP) regulation. Managing hypertension in patients with OH presents a significant clinical challenge, particularly in frail older adults who are also prone to falls. Hypertension treatment is often suboptimal in this population due to concerns over the potential increased risk of falls associated with treatment. However, current clinical guidelines provide limited guidance on managing this complex issue. This review explores the pathophysiology of hypertension and OH, reviews existing guidelines, and examines the evidence surrounding hypertension management in patients with OH. Additionally, we provide an overview of research focused on frail older adults and offer expert-opinion-based recommendations for the management of hypertension and OH in routine clinical practice.

## Graphical abstract

**Figure:**
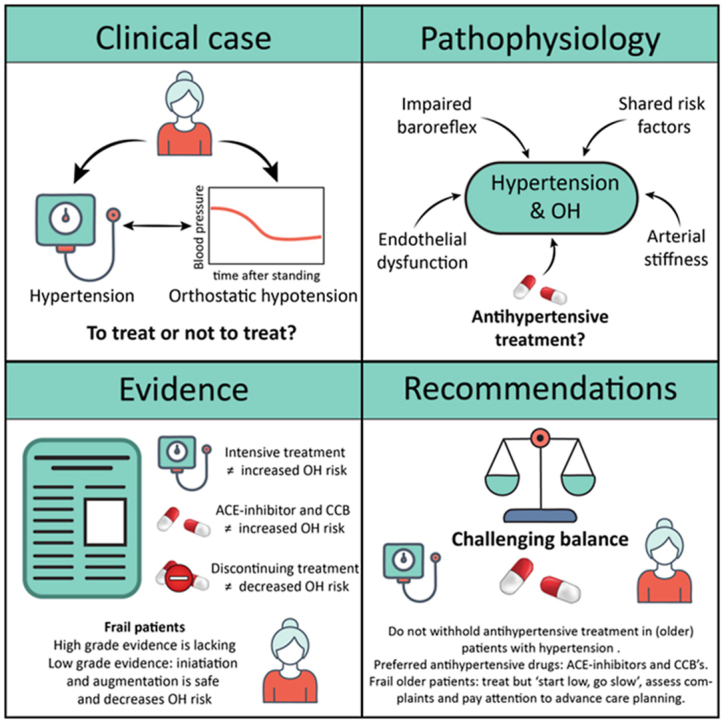

## Clinical case presentation and introduction

An 83-year-old man with the presented medical history and concomitant medication is referred to the geriatric outpatient clinic for recurrent falls (Box 1). During physical examination, hypertension and evident orthostatic hypotension (OH) stand out.

**Box 1** **Clinical case.**

Hypertension and OH are common in the older population, with an increasing prevalence with age.^1^^,^^2^ These conditions have been associated with several health concerns, including increased risk of falls, cardiovascular disease, stroke, cognitive decline, dementia, and death.^3^, ^4^, ^5^, ^6^, ^7^, ^8^ Both hypertension and OH could be seen as a result of impaired blood pressure (BP) regulation and are highly intertwined.^9^ Approximately 10% of patients with hypertension experience OH, and up to 70% of patients with OH are also diagnosed with hypertension.^10^^,^^11^ Yet, treatment of patients with both hypertension and OH poses a challenging balance, particularly in frail older patients reporting falls. Hypertension treatment in patients with OH is often suboptimal due to an anticipated increased risk of falls.^12^^,^^13^ A recent, but limited, study showed that primary care physicians more often chose to deprescribe antihypertensive treatment in patients with OH compared to patients without OH.^14^ Perhaps somewhat contradictory, a recent systemic review and individual participant-based meta-analysis indicated that treatment of hypertension reduces the prevalence of OH.^15^

The patient in question presents suffers from recurrent falls, with evident orthostatic hypotension (OH) as a potential causal or contributing factor. This situation presents a clinical dilemma, particularly given the patient's history of hypertension and current use of the antihypertensive agent enalapril. The key question arises: should antihypertensive therapy be tapered in response to the presence of OH, or should hypertension management be intensified? Additionally, determining appropriate target blood pressure values for this patient, as well as selecting the most suitable antihypertensive medications, requires careful consideration. It is crucial to evaluate the risks posed by OH and to factor in the patient's level of frailty when making treatment decisions. Achieving an optimal balance in this context is a significant clinical challenge.

In this review, we discuss the pathophysiology of the combination of hypertension and OH, present current guidelines, review current evidence on hypertension treatment in patients with OH, give an overview of research specifically in frail older patients, and conclude with hands-on expert opinion-based recommendations for management of hypertension and OH in older adults. See Fig. 1 for a graphical abstract of our findings.

## Search strategy and selection criteria

We performed a narrative review of existing literature. PubMed (from 1990 to June 1st 2024) was used to search for relevant published articles using the search terms: “Orthostatic hypotension”, AND “Hypertension” AND “Antihypertensive treatment”. We focused on peer-reviewed systematic reviews, meta-analyses and randomized controlled trials. Further, we looked specifically at articles focused on older patients (aged ≥60 years). The references of the identified articles were searched for other relevant studies. Only papers published in English were reviewed. Articles were included on the basis of relevance to the scope of this review. The most recent guidelines from different health care associations (American Hypertension Association (AHA), European Society of Hypertension (ESH), International Society of Hypertension (ISH), National Institute for Health and Care Excellence (NICE), Dutch College of General Practitioners (DCGP/NHG)) were used to present BP targets and recommendations on OH.^16^, ^17^, ^18^, ^19^, ^20^

## Pathophysiology

BP is regulated by two systems: first the RAAS-system; responsible for overall BP, electrolyte and fluid regulation and second autonomic mechanisms, including the baroreceptor reflex system, responsible for maintaining arterial pressure throughout (patho)physiological sudden changes, such as postural change, exercise, or haemorrhage.^21^ In particular, the baroreceptor reflex system plays a role in OH.

When standing up, 500–700 ml of blood pools in the abdomen and pelvic region and lower limbs due to gravitational forces,^22^ and muscle activation during active standing causes arterial vasodilation. In older patients the calf muscle vein pump is reduced which further contributes to venous pooling in the lower limbs.^23^ Together these mechanisms cause a quick reduction of venous return to the heart, resulting in reduced preload and stroke volume. The subsequent loss of arterial pressure is sensed by baroreceptors, which consequently reduce parasympathetic tone, followed by an increase in sympathetic tone. This causes an increase in heart rate and peripheral vasoconstriction, which directly increases BP.^24^

In patients with OH, the baroreflex function falls short, resulting in a sustained drop in BP.^9^ The sensitivity of the baroreflex decreases with age,^25^ and is also disrupted by the presence of hypertension and arterial stiffness.^26^^,^^27^ Impaired baroreflex function also results in increased BP variability,^28^ which is associated with adverse outcomes.^29^

Another possible pathophysiological connection is through endothelial dysfunction and arterial stiffness, factors associated with both hypertension and orthostatic hypotension. Endothelial dysfunction can be triggered by shear stress and is involved in the initiation and development of vascular inflammation, vascular remodelling and atherosclerosis, resulting in further increased arterial stiffness and peripheral blood pressure.^30^ Markers of these pro-inflammatory changes and endothelial dysfunction are also associated with an increased risk of OH.^31^^,^^32^ Arterial stiffness is inversely associated with baroreceptor sensitivity and can therefore contribute to the drop in BP after standing up. The aforementioned mechanisms are all interrelated and involved in impaired BP regulation.

Alongside these age and hypertension related effects baroreflex function can also become impaired due to autonomic failure in several neurodegenerative diseases, most frequently in Parkinson disease or Parkinson-like diseases. In this review, we will not focus on autonomic failure as a cause of OH.

Another explanation for the link between hypertension and OH is the presence of shared risk factors, including diabetes, chronic kidney disease, atrial fibrillation, heart failure or stroke.^33^, ^34^, ^35^, ^36^, ^37^, ^38^, ^39^ For example, both atrial fibrillation and heart failure can hamper ventricular filling, while diabetes can lead to autonomic neuropathy, both increasing the risk for OH. Both atrial fibrillation and heart failure are treated with medication that increases the risk of OH, including beta-blockers and diuretics.^40^

The triad of hypertension, diabetes and orthostatic hypotension warrants additional attention, as they are strongly interconnected and associated with adverse events.^36^^,^^41^^,^^42^ Diabetes and hyperglycaemia contribute to the development of hypertension due to inappropriate RAAS activation, endothelial damage and arterial stiffness.^43^ Furthermore, patients with diabetes are at increased risk of OH, both through hypertension and possible autonomic neuropathy.^44^ Orthostatic hypotension in patients with diabetes is associated with falls and cognitive decline.^45^^,^^46^ Therefore, managing hypertension and diabetes to reduce the likelihood of OH may be particularly crucial in this population.

Lastly, patients with hypertension more easily meet the OH definition. This definition is based on an absolute reduction in mmHg BP (≥20 mmHg SBP or ≥10 mmHg DBP),^47^ which is met with a smaller relative reduction in patients with a higher supine BP.^48^ For this reason, in hypertensive patients, a systolic cut-off value of 30 mmHg has been suggested.^49^

The interplay between hypertension, orthostatic hypotension (OH), and frailty is strongly linked to both cognitive and physical impairment in older adults.^50^, ^51^, ^52^ Hypertension, particularly in its chronic form, contributes to vascular damage, reduced cerebral perfusion, and microvascular dysfunction, all of which increase the risk of cognitive decline and dementia.^53^ In frail individuals, who already experience diminished physiological reserves and increased vulnerability, this cognitive impairment can be further exacerbated by OH. Recurrent OH events, common in frail and hypertensive patients, can compound cognitive decline by reducing blood flow to critical brain regions.^54^ Moreover, these hemodynamic fluctuations can worsen physical impairment, as frail individuals already experience reduced muscle strength, balance issues, and limited mobility.^55^ The combined effect of hypertension and OH thus accelerates physical decline, increasing the risk of falls and injury, while also heightening cognitive vulnerability.^56^^,^^57^ This triad of hypertension, OH, and frailty creates a feedback loop of deteriorating cognitive and physical function.

In conclusion, there are several (patho)physiological mechanisms that play a role in the complex relation between hypertension and OH.

## Current guidelines

(Inter)national guidelines are used to initiate or optimize treatment in patients with hypertension and some guidelines provide recommendations on OH assessment and treatment of hypertension in (older) patients with OH.

Table 1 gives an overview of different current guideline recommendations on general BP targets, BP targets in older adults, measurement of OH, and treatment of hypertension in patients with OH.^16^, ^17^, ^18^, ^19^, ^20^

**Table 1:** Hypertension guidelines, blood pressure (BP) targets and recommendations on OH measurement.

Hypertension guideline	General BP targets	BP targets for older adults	Recommendation on OH measurement	Recommendation on hypertension treatment in patients with OH
AHA guidelines (2017)^21^	Target BP <130/80 mmHg	Target SBP <130 mmHg for non-institutionalized ambulatory community-dwelling older adults ≥65 years.	Change in BP from seated to standing position should be measured to detect OH, especially if symptoms are present or in older patients.	Improved BP control in older patients does not exacerbate OH and has no adverse impact on risk of injurious falls.
Dutch guidelines (NHG) (2019)^20^	Target SBP <140 mmHg, <130 mmHg if tolerated	Target SBP <150 mmHg and if tolerated <140 mmHg, for adults ≥70 years without physical frailty.	Not given	Not given.
ESH guidelines (2023)^17^	Target BP <130/80 mmHg	Target SBP 130–139 mmHg, target DBP <80 mmHg if tolerated for persons ≥65 years.	In older persons (>65 years of age), diabetic patients, patients with neurodegenerative disorders, or with symptoms suggesting OH, BP should also be measured 1 and 3 min after standing.	Reduction of treatment can be considered in the presence of (severe) OH.
ISH guidelines (2020)^18^	Target BP <130/80 mmHg	Target BP < 140/90 mmHg if tolerated for persons ≥65 years.	OH measurement if symptoms of OH are present or in older patients.	Not given.
UK guidelines (NICE) (2019)^19^	Target BP <140/90 mmHg	Target BP < 150/90 mmHg for adults >80 years.	Measure BP after 1 min of standing in people with symptoms of OH, including falls or postural dizziness	Manage appropriately.

Abbreviations: AHA; American Heart Association, BP; Blood pressure, ESH; European Society of Hypertension, ISH; International Society of Hypertension, NHG; Nederlands Huisartsen Genootschap (Dutch College of General Practitioners), NICE; the National Institute for Health and Care Excellence, OH; Orthostatic Hypotension, SBP; Systolic Blood Pressure, UK; United Kingdom.

In conclusion, almost all hypertension guidelines recommend OH measurement in patients with hypertension. However, there are limited and non-specific recommendations on hypertension treatment in patients with OH, with no consensus on whether treatment should differ in patients with OH compared to patients without OH. The European guideline mentions antihypertensive treatment reduction can be considered, whilst the AHA guidelines states that there is no increased risk of developing OH through antihypertensive treatment. British guidelines recommend managing the combination of OH and hypertension appropriately but give no further explanation. These differences show the medical dissensus and highlight an important gap in clinical knowledge.

## Current evidence

### Hypertension treatment and risk of orthostatic hypotension

To make informed clinical decisions on antihypertensive treatment in hypertensive patients with OH, it is important to explore current research on the role of BP targets and antihypertensive treatment in the risk of developing OH.

#### Hypertension treatment and BP targets

Several large, randomized trials have looked at the difference between intensive vs standard BP targets and the incidence of OH.^58^, ^59^, ^60^, ^61^, ^62^ In 2021, Juraschek et al. published a systematic review and participant-based meta-analysis including five studies and combined total of 18,466 participants focusing on BP treatment goal (intensive vs standard BP control; intensive ranging from <150 mmHg SBP to <120 mmHg SBP).^15^ This review showed that all trials comparing different BP goals reported lower odds of OH in the intensive treatment group, with a pooled OR (95% CI) of 0.93 (0.86–0.99). It is important to note that these studies predominantly included younger and fitter patients (mean age in all studies was <65 years). The interaction for age (≤75 vs > 75 years) was non-significant, and risk of OH (OR; 95% CI of 0.92; 0.79–1.09) was similar in patients aged >75 years (n = 2895).

The systematic review also included four placebo-controlled trials, of which the HYVET study showed a lower risk of OH in the treatment group (OR; 95% CI was 0.74; 0.56–0.99),^63^ while the other three showed no significant difference between the treatment and control group.^36^^,^^64^^,^^65^ Interestingly, the HYVET study focused specifically on patients aged 80 years and older. However, patients with secondary hypertension, cognitive disorders or gout were excluded, resulting in an overall healthier study population than the general older population.

Another individual participant data meta-analysis, including nine trials with 29,235 participants, showed that antihypertensive therapy reduced risk of CVD or all-cause mortality regardless of baseline orthostatic hypotension.^66^ Finally, a systemic review and meta-analysis reporting on the association between antihypertensive treatment and adverse events found no evidence that antihypertensive treatment is associated with falls, however this was not prospectively measured.^67^

In conclusion, more intensive blood pressure treatment reduced the risk of orthostatic hypotension (OH), with consistent effects observed in older subgroups. More intensive BP management also lowered the risk of cardiovascular disease (CVD) and was not associated with an increased incidence of falls in cross-sectional analyses.

A possible explanation for the lower prevalence of orthostatic hypotension (OH) in patients undergoing hypertension treatment may be the improvement in baroreceptor function.^68^^,^^69^ Alternatively, it could be due to the requirement for a relatively larger reduction in blood pressure to meet the diagnostic criteria for OH.^48^

#### Different antihypertensive classes

A number of studies have reported on the different antihypertensive classes and the risk of OH.^49^ A systematic review and meta-analysis on drug-induced OH,^40^ showed that antihypertensive classes interfering with sympathetic compensatory responses (alpha- and betablockers) are more strongly associated with OH than other antihypertensive classes. Use of beta-blockers was associated with a strikingly increased risk of OH; OR (95% CI) of 7.8 (2.5–24.0). Calcium-channel blockers (CCB), angiotensin converting enzyme (ACE) inhibitors, and angiotensin receptor blockers (ARB) were not associated with an increased risk of OH. ACE-inhibitors/ARB's and CCB's lower BP by affecting the Renin Angiotensin Aldosterone-System and vasodilation, but have a limited effect on the sympathetic compensatory response to standing up, which is important in OH development.^40^ Diuretics, another important antihypertensive drug class, were not included in this meta-analysis, but several observational studies have reported on this association and have shown a small increased risk.^70^, ^71^, ^72^, ^73^ Diuretics, especially loop diuretics, can cause volume depletion and contribute to the development of OH. While thiazide diuretics can also cause volume depletion, this effect is predominantly short-term making them a safer option in hypertension treatment, especially in older adults.^74^^,^^75^

In general, observational studies showed an increased risk of OH in most antihypertensive classes.^70^^,^^76^ However, an observational design limits assessment of causality and this association could also be due to increased OH risk as a result of hypertension, rather than the medication prescribed for hypertension. To draw conclusions on causal relationships, randomized controlled trials are far more reliable.

#### Discontinuing antihypertensive treatment

As stated before, primary care physicians tend to withhold or even deprescribe antihypertensive treatment in older patients with hypertension and OH.^14^ Two studies investigated the effect of discontinuing antihypertensive medication in patients with hypertension on risk of OH. The largest trial included 978 patients (aged 60–80) and revealed that antihypertensive discontinuation increased OH symptoms and related events such as falls.^77^ In contrast, the DANTE trial (N = 162) reported a decrease in prevalence of OH after deprescribing antihypertensive therapy in older patients with mild cognitive impairment, however in the intention to treat group, there were no differences.^78^

### Antihypertensive treatment and orthostatic hypotension in older, frail, patients

As stated above, post-hoc analyses of HYVET and SPRINT did not find an interaction between the benefits of antihypertensive treatment and frailty and concluded that treatment goals should be similar. However, frail patients were excluded from both studies, as well as in most other clinical trials.^79^ The Irish Longitudinal Study on Ageing (TILDA) included a more representative, truly frail, sample and found that frailty had no adverse impact of events caused by antihypertensive treatment (including syncope and falls). However, this was a non-randomized longitudinal cohort study.^80^ Claassen et al. published a small non-randomized and unblinded intervention trial (n = 14), in which the initiation or augmentation of antihypertensive therapy (mostly ARB or CCB) to achieve a target systolic BP < 140 mmHg did not increase prevalence of OH in frail older hypertensive adults.^81^ In line with this observation, a recent systematic review and meta-analysis concluded that in older patients (>65 years) antihypertensive treatment initiation or augmentation decreased OH prevalence, although with relative low grade of evidence.^82^

## Recommendations

### Antihypertensive treatment and OH

In conclusion, currently available evidence demonstrates that (intensive) antihypertensive treatment does not increase the risk of OH in older patients. Therefore, a perceived risk of OH should not be a reason to withhold or deprescribe treatment in older patients. In general, without patient-specific contra-indications or disease indicated preferred drugs, the overall preferred antihypertensive classes are ACE-inhibitors, ARB's and CCB's for older patients, as alpha- and beta-blockers seem to increase risk of OH, and diuretics might.

**Fig. 2:**
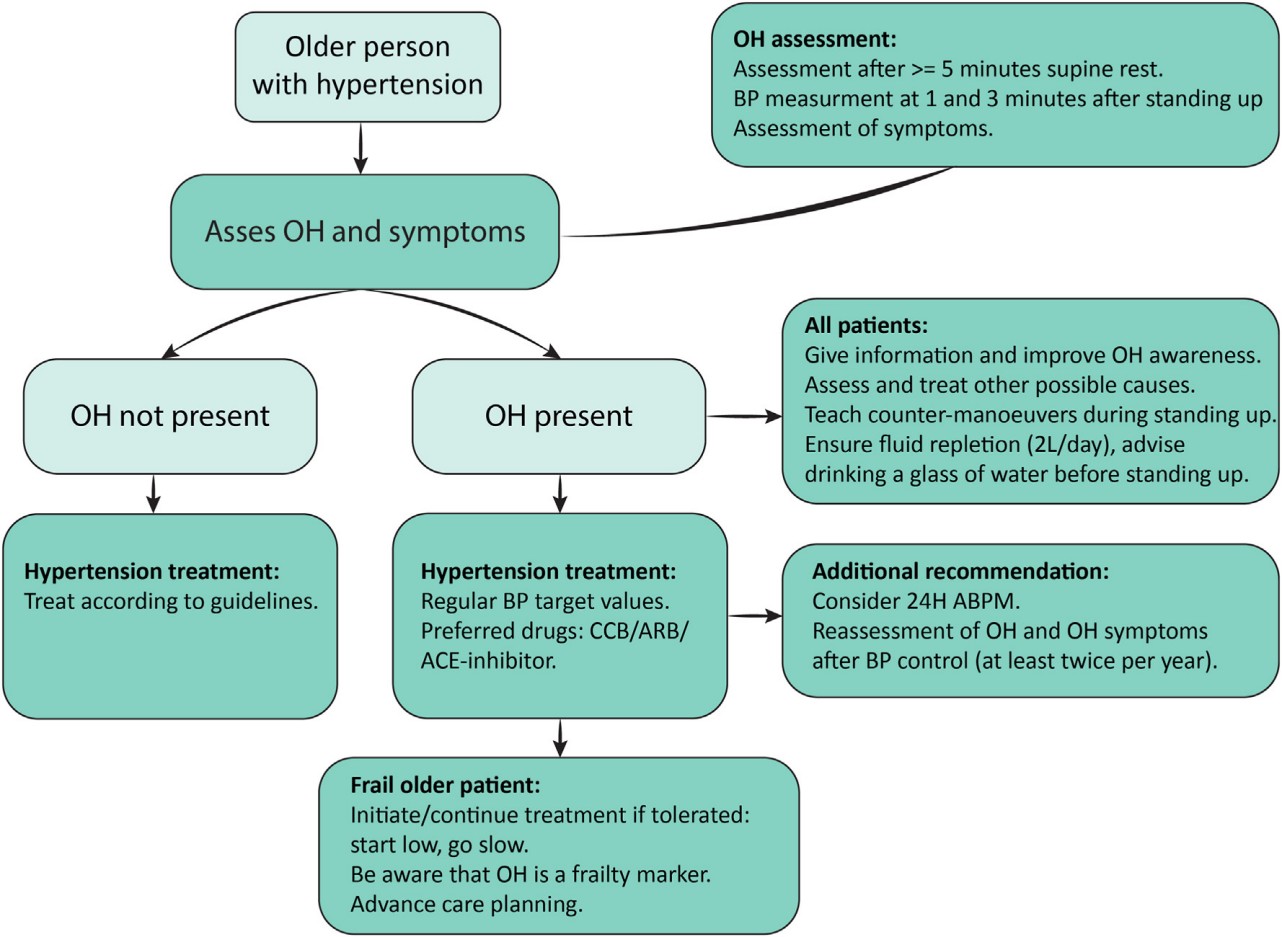
OH Assessment and hypertension treatment in older patients with hypertension. Abbreviations: ABPM; ambulatory blood pressure measurement, ACE: angiotensin converting enzyme, ARB: angiotensin receptor blocker, CCB: calcium channel blocker, OH: orthostatic hypertension.

Fig. 2 visualizes a suggested work-up in older patients with hypertension: in all patients with OH, in addition to optimal hypertension treatment with an ACE-inhibitor or CCB, information on OH and explanation of possible counter-manoeuvres, such as standing up slowly, crossing the legs and tightening the glute muscles should be given, and volume repletion should be ensured.

Furthermore, other underlying causes of OH should be treated optimally, including a medication review focusing on OH-aggravating drugs, such as tricyclic antidepressants and alpha-blocking urologicals, followed by appropriate deprescribing of redundant pharmacological agents or switching to an alternative with fewer side-effects.^40^^,^^83^ As an example, replacing an alpha-blocker with a 5-alpha-reductase inhibitor in case of BPH could be a solution for patients with OH.

### Frail patients

The question remains if hypertensive frail older patients with and without OH, still benefit from antihypertensive treatment. This has been thoroughly debated, as several articles tried to articulate recommendations for this population. The overall consensus is to prescribe antihypertensive medication if tolerated and when life-expectancy is sufficient, but to do so with caution.^84^, ^85^, ^86^, ^87^ Special consideration should be given to patients with OH and falls, who will often classify as frail. Orthostatic hypotension is an established risk factor for falls, and many successful RCTs that have performed multifactorial fall risk assessments have included assessment and treatment of OH.^3^^,^^88^^,^^89^ Evidence regarding the tapering of antihypertensive medication in frail patients with the complex combination of hypertension, OH and falls is still lacking. For frail patients presenting with hypertension and OH, an individualized approach with shared decision-making is warranted. Lifestyle options, time to benefit and life expectancy should be taken into account when initiating BP treatment. When BP treatment is initiated, medication should be started at the lowest effective dose, after which the dose is titrated to reach target BP levels (‘start low and go slow’).^53^^,^^85^ Development of OH and its symptoms should be monitored during clinical visits. A 24-h Ambulatory BP Measurement (ABPM) can be considered for additional insights into overall BP control and systolic BP dips that could cause symptoms.^90^^,^^91^

### Presented clinical case

It is plausible that the reason for referral in our patient, an 83-year-old male with recurrent falls, is the result of OH. Therefore, optimal treatment should minimize the risk of OH (Box 2). Non-pharmaceutical advice and information on OH should always be given. Replacing tamsulosin with a 5-alpha-reductase inhibitor might reduce risk of OH. In this case, it would be valid to intensify the antihypertensive treatment in order to lower the increased cardiovascular risk and risk of OH with a low dose CCB or increase in enalapril dosage as preferred antihypertensive drug options. It is key to start low and go slow when changing antihypertensive treatment. Target values for this patient are below 140 mmHg systolic but only if tolerated. Follow-up should be performed by the same health professional to evaluate OH symptoms and possible side-effects of initiated therapy.

**Box 2** **Clinical case recommendations.**

### Conclusion

Hypertension and OH are two strongly intertwined conditions, partly due to the impaired baroreflex function, and both are highly prevalent in older patients. Despite common belief, more intensive hypertension treatment does not seem to induce OH. Preferred antihypertensive agents are CCB's and ACE-inhibitors/ARB's to minimize risk of OH. There is no reason to modify this advice in older hypertensive patients. In frail older patients, thorough research is missing and the overall consensus is to treat using a personalized treatment plan and shared decision making.

## Contributors

Julia HI Wiersinga: conceptualization, investigation, methodology, visualization, writing–originial draft. Sofie Jansen: conceptualization, investigation, methodology, writing–originial draft. Mike J.L. Peters: supervision, writing–review & editing. Hanneke F.M. Rhodius-Meester: supervision, writing–review & editing. Marijke C. Trappenburg: supervision, writing–review & editing. Jurgen Claassen: supervision, writing–review & editing. Majon Muller: supervision, writing–review & editing.

## Declaration of interests

We declare the following interests. Hanneke-Rhodius Meester has received grants from TKI-Health Holland (DAILY, project number LSHM19123-HSGF), Alzheimer Nederland (InterACT, project number WE.08-2022-06), ZonMW (Memorabel Dementia Fellowship 2021, projectnumber 10510022110004), Horizon 2022 EU (PROMINENT, project number 101112145), Combinostics. Jurgen Claassen receives annual research budget form Radboud UMC, and research grants (paid to institution) from Alzheimer Nederland, ZonMW (NWO), Gieskes Strijbis Fund. He has given presentations at Eisai, BMS, Lilly, Several small Dutch conference organisers, is on the scientific advisory board of Alzheimer Nederland and associate editor at JCBFM. Majon Muller has given presentations for ViV–HIV and aging, CarVascGer congress–Heart failure medication and fall risk, Heart failure master class—GDMT in older heart failure patients, Dutch Lipids Experience—Statin treatment in the old. She is on the supervisory board for stichting Hartekind and board member at Dutch Internal Medicine association—NIV.
